# Detection of Japanese encephalitis virus in *Culex tritaeniorhynchus* following the 2024 Noto Peninsula earthquake, Japan

**DOI:** 10.1016/j.parepi.2026.e00539

**Published:** 2026-08-23

**Authors:** Kota Mochizuki, Yosaburo Oikawa, Manabu Murakami

**Affiliations:** Department of Medical Zoology, Kanazawa Medical University, Japan

**Keywords:** Japanese encephalitis virus, *Culex tritaeniorhynchus*, 2024 Noto peninsula earthquake

## Abstract

Japanese encephalitis virus (JEV) is a mosquito-borne zoonotic flavivirus transmitted primarily by *Culex* (*Cx.*) *tritaeniorhynchus* and the risk of JEV transmission is influenced by environmental and ecological conditions. Large-scale natural disasters can substantially alter local environments and mosquito habitats, potentially affecting the circulation of arboviruses. Following the January 1, 2024, Noto earthquake, the surrounding environment in the region changed drastically.

In this study, we investigated the prevalence and genetic characteristics of JEV in mosquito populations collected before and after an earthquake in the affected areas and its surrounding region. The climatic and environmental conditions during the sampling periods were also compared to assess their potential contribution to changes in JEV positivity.

Despite the lack of statistical significance, JEV was detected exclusively in post-earthquake mosquito samples, and all the detected strains belonged to genotype Ib (GI-b). A phylogenetic analysis further revealed the presence of distinct clades within GI-b, suggesting that multiple independent introduction events had occurred in the past.

The detection of multiple sequences of JEV GI-b in mosquitoes collected after an earthquake warrants particular attention, as ongoing post-disaster reconstruction and associated environmental changes may further influence viral circulation, underscoring the need for continued and careful surveillance.

## Highlights

• Japanese encephalitis virus (JEV) was detected in post-earthquake mosquitoes.
• All JEV strains detected were genotype Ib.
• Different JEV clades coexisted within the same year and site.

## Graphical abstract

**Figure:**
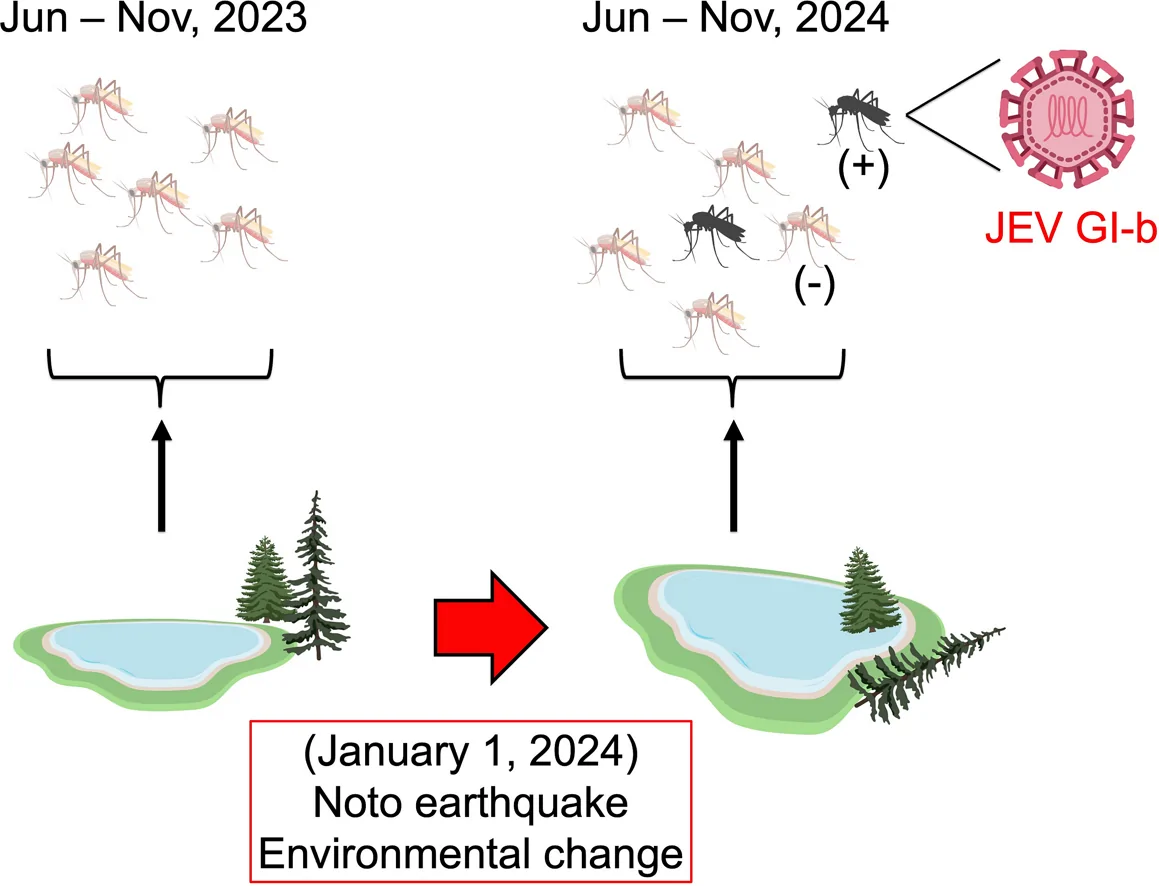

## 1 Introduction

**Fig. 1:**
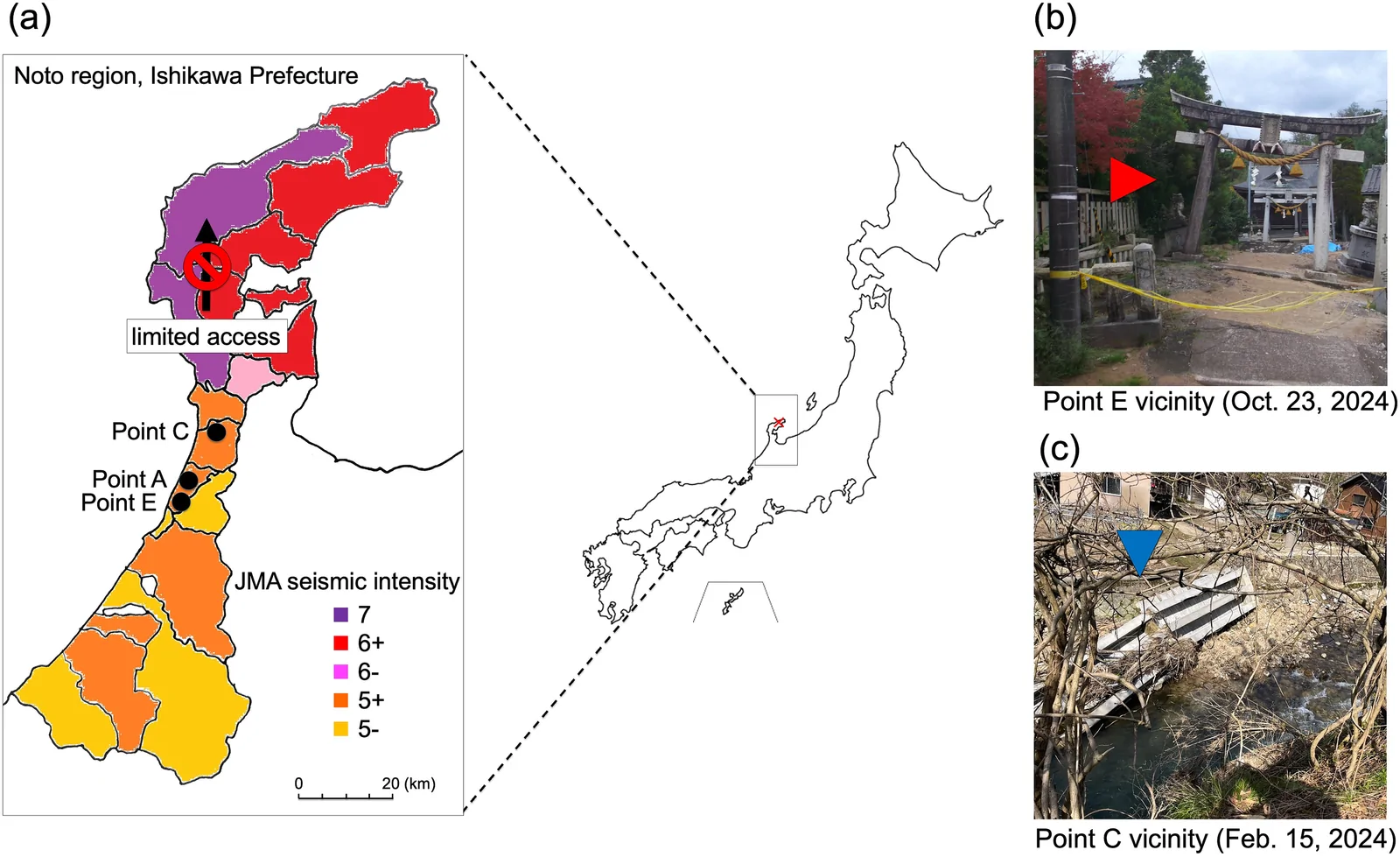
Trap sites of *Cx. tritaeniorhynchus* in Ishikawa Prefecture. (a) × indicating the epicenter of the 2024 Noto earthquake on January 1. Colors indicating JMA seismic intensity (7: purple - 5-lower: light orange). (b) Vicinity of Point E, showing earthquake-induced tilting of the shrine gate and surrounding ground, with cracking (red arrowhead). (c) Vicinity of Point C, showing collapsed riverbank and damaged revetment (blue arrowhead). (For interpretation of the references to colour in this figure legend, the reader is referred to the web version of this article.)

Japanese encephalitis virus (JEV) is a mosquito-borne zoonotic flavivirus that remains a major cause of viral encephalitis in Asia, affecting thousands of individuals annually (Morita et al., 2015). JEV circulates in a mosquito-borne enzootic cycle involving mosquitoes, birds, and pigs, with humans as incidental dead-end hosts (Weaver and Reisen, 2010). The primary vector in endemic regions of Asia, including Japan, is *Culex* (*Cx.*) *tritaeniorhynchus* (Edache et al., 2025; Pearce et al., 2018). The epidemiology of Japanese encephalitis (JE) is closely linked to environmental and ecological conditions that affect mosquito vectors and amplifying hosts, and changes in these factors have been implicated in the resurgence and spread of JEV (Mackenzie et al., 2004). In a spatial epidemiological study in China, village level clustering of JE cases was associated with environmental factors such as distance from roads and water-related indices that may contribute to variation in infection risk (Liu et al., 2024). The recent human JE cases in Chiba, Japan, suggest that JE can occur in areas with few or no reported cases in recent decades (Yoshizawa et al., 2025). In Ishikawa Prefecture, including the Noto region (Fig. 1), surveillance has documented persistent JEV circulation in *Cx. tritaeniorhynchus* populations, although the mosquito infection rates varied among sites and years during previous surveys (Murakami et al., 2017). Given the dynamic changes in the spatial spread of JEV, assessing the infection status of vectors is essential for predicting their future distribution. Regarding the method used to survey the prevalence of JEV in *Cx. tritaeniorhynchus* populations, Centers for Disease Control (CDC) miniature traps baited with CO_2_ are commonly employed in mosquito surveillance and have been shown to efficiently collect *Culex* mosquitoes (McNamara et al., 2021).

JEV is classified into five genotypes (I-V), and their temporal and geographic distributions have shifted over time (Xia et al., 2023; Uchil and Satchidanandam, 2001). In Japan, JEV genotype I (GI) has gradually replaced the historically dominant GIII (Schuh et al., 2014). Several molecular and experimental studies have reported that GI strains exhibit higher replication efficacy than GIII strains under experimental condition (Fan et al., 2019; Xiao et al., 2018). Among the recent emerging trends, a GIV-associated outbreak occurred in Australia in 2022 (Waller et al., 2022), where relatively low JE vaccine coverage may have contributed to limited population immunity (Howard-Jones et al., 2022). In addition, GV has been sporadically reported in East Asian regions outside Japan (Woo et al., 2020; Li et al., 2025), raising concerns because currently available JE vaccines may provide reduced cross-protection against some GV strains (Cao et al., 2016). Thus, longitudinal monitoring of JEV is essential for understanding viral evolution and potential shifts in transmission patterns.

We conducted continuous monitoring of JEV prevalence in mosquito pools in Ishikawa Prefecture before and after the 2024 Mw 7.5 Noto earthquake on January 1. Natural disasters, such as earthquakes and flooding, can substantially alter environmental conditions that influence vector ecology (Watson et al., 2007; Shokri et al., 2020). For instance, the 2010 earthquake in Haiti triggered rapid urbanization and land-use changes that modified the suitability of habitats for mosquito species, including *Culex* and *Aedes*, likely influencing their ecology and potentially increasing the risk of emerging vector-borne diseases in post-disaster environments (Samson et al., 2015). Although increases in the incidence of vector-borne diseases have been reported following major earthquakes, the current literature primarily focuses on human cases and environmental risk factors, and relatively little attention has been paid to direct pathogen detection in mosquito vectors in post-earthquake settings (Zeyrek et al., 2023; Mavrouli et al., 2023).

The objective of this study is to evaluate the chronological impact of the 2024 earthquake on JEV dynamics by characterizing its temporal detection patterns and molecular characteristics. To achieve this, we combined systemic longitudinal field surveillance with statistical comparisons and phylogenetic evaluation. Given the scarcity of longitudinal vector surveillance studies encompassing pre- and post-disaster periods, this study provides valuable insights into the impact of major natural disasters on JEV circulation in mosquito populations.

## 2 Materials and methods

### 2.1 The study area

On January 1, 2024, a powerful earthquake with a magnitude of 7.5 hit the Ishikawa Prefecture in Japan. This earthquake was named the Noto Peninsula Earthquake and caused devastating damage to Suzu, Wajima, Nanao city, and Noto, Shika town. The northern area near the epicenter was inaccessible because of the severe road damage and traffic restrictions. All collection sites (points A, C, and E) were located in the earthquake-affected area and its surrounding region (Fig. 1).

Longitudinal mosquito surveys with weekly collections were carried out in pig farms and rice field areas: point A Mori, Kahoku city, A: 36°43′ 07.53 N, 136°42′ 49.34E; point C Hodatsushimizu town, Hakui-gun, C: 36°49′ 48.65 N, 136°46′ 17.93E; and point E, Uchihisumi, Kahoku city, E: 36°42′ 07.63 N, 136°41′ 51.13E (Fig. 1). Points A and E, Kahoku City, are lowland areas with hilly terrain and alluvial plains of coastal sand dunes 12 km south–north and 12 km east–west, surrounded by rice fields. Approximately 53% of the area was agricultural land, 25% was forest wilderness, and 18% was residential land. Point C, Hakui-gun, is a lowland area with hilly terrain and an alluvial plain, 11 km long and 11 km wide, with abundant rice cultivation. The area consisted of approximately 46% agricultural land, 25% forest or wilderness, and 11% residential land.

### 2.2 Mosquito collections and identification

Adult mosquitoes were collected once a week using a single Centers for Disease Control (CDC) miniature light traps (Model 512; John W. Hock Co., Gainesville, FL, USA) per site, which was baited with 1 kg of dry ice (CO_2_) and set from 5:00–6:00 p.m. to 9:00–10:00 a.m. the following morning between June and November. Mosquitoes were collected from traps each morning. The collected samples were placed in an insulated container with cold packs, brought to the laboratory, and frozen upon arrival. Mosquitoes were removed from the container, and each specimen was identified using a stereomicroscope.

Specimens were pooled according to species, date, and trap location. Female *Cx. tritaeniorhynchus* were identified morphologically and labeled based on the date and geographic region of their capture. Pools contained up to 35 mosquitoes in 15 mL plastic containers. The pooled female *Cx. tritaeniorhynchus* were homogenized in 3 mL of Minimum Essential Medium Alpha (Sigma-Aldrich, Germany) using a mixer, filtered using a combined 0.8/0.2-μm filter and stored at −80 °C until viral screening.

### 2.3 Weather and seismic intensity of earthquake, statistical analysis

Meteorological data for Ishikawa Prefecture were obtained from the publicly available database of the Japan Meteorological Agency (JMA) (https://www.data.jma.go.jp/stats/etrn/index.php). The seismic intensity of the 2024 Noto Peninsula earthquake was obtained from the publicly available database of the JMA (https://www.jma.go.jp/bosai/map.html#12/&contents=estimated_intensity_map&id). A generalized linear mixed-model (GLMM) with a binomial distribution was performed. JEV infection status was entered as the dependent variable. Fixed effects included pre- or post-earthquake status, mean temperature, precipitation, and the number of captured mosquitoes. All continuous explanatory variables were standardized prior to analysis. Site and year were included as random effects. For the multi-year analysis, we combined our data with the raw data from a previous study (Murakami et al., 2017). To ensure the comparability between different study periods, overlapping weeks (Weeks 23–43) were used for the analysis. All statistical analyses were conducted using R (version 4.3.3) with the lme4 (Bates et al., 2015), and tidyverse packages (Wickham et al., 2019). Graphical abstract was created using BioRender (https://biorender.com/). Owing to rain and wind conditions, CDC trapping could not be conducted in July 2024.

### 2.4 RNA extraction of pooled mosquitoes and RT-PCR

Frozen mosquito homogenates were subjected to RNA extraction using the QIAamp Viral RNA Mini Kit (Qiagen, Hilden, Germany), according to the manufacturer's instructions. RT-PCR was performed in 10 μL using a SuperScript III One-Step RT-PCR system with Platinum *Taq* DNA polymerase (Invitrogen, USA; catalog no. 12574–018) as per the manufacturer's protocol with the following conditions: 0.5 μM of each MAMD (5′- AACATGATGGGRAARAGRGARAA −3′) and cFD2 (5′- GTGTCCCAGCCGGCGGTGTCATCAGC -3′) primers targeting nonstructural protein 5 (NS5) (Scaramozzino et al., 2001), JEV-Ef (5′- TGYTGGTCGCTCCGGCTTA −3′) and JEV-Er (5′- AAGATGCCACTTCCACAYCTC -3′) primers targeting envelope (E) protein (Gao et al., 2013), respectively, and 1 μL of the extracted RNA. RT-PCR was performed under the following conditions: 53 °C for 30 min, followed by initial denaturation at 94 °C for 2 min, 40 cycles of 94 °C for 15 s, 53 °C for 30 s, and 68 °C for 90 s, and a final extension at 68 °C for 5 min. The amplicons produced by the RT-PCR were confirmed by running the products on 1.5% (*w*/*v*) agarose gels supplemented with Midori Green Advance (Nippon Genetics, Tokyo, Japan). The minimum infection rate (MIR) was calculated using the formula: MIR = (number of positive pools)/(number of tested mosquitoes) × 1000.

### 2.5 Nucleotide sequencing of Flaviviruses

To sequence the nucleotides of the amplicon of NS5 and the E protein of flaviviruses, RT-PCR-positive samples were purified using the NucleoSpin® Gel and PCR Clean-up kit (MACHEREY-NAGEL, Germany) according to the manufacturer's protocol. The purified PCR amplicons were subjected to Sanger sequencing. Sequencing reactions were performed by the institutional service using BigDye™ Terminator v3.1 (Thermo Fisher Scientific, USA) on an Applied Biosystems 3500xL Genetic Analyzer (Thermo Fisher Scientific, USA). All sequence data in this study have been deposited in GenBank under the accession numbers LC915187-LC915198 and LC934768-LC934779.

### 2.6 Phylogenetic analysis

The NS5 and E protein gene sequences of the RT-PCR-positive samples were analyzed by BLAST-N search, and DNA alignment was performed using MUSCLE (Edgar, 2004). Phylogenetic trees were constructed using MEGA12 (Kumar et al., 2024) based on the maximum likelihood and neighbor-joining methods after estimation of genetic distance using the Tamura-Nei method (Tamura and Nei, 1993). A bootstrapping test was performed with 1000 duplicates.

## 3 Results

### 3.1 Collection of *Culex tritaeniorhynchus* and climate conditions

**Fig. 2:**
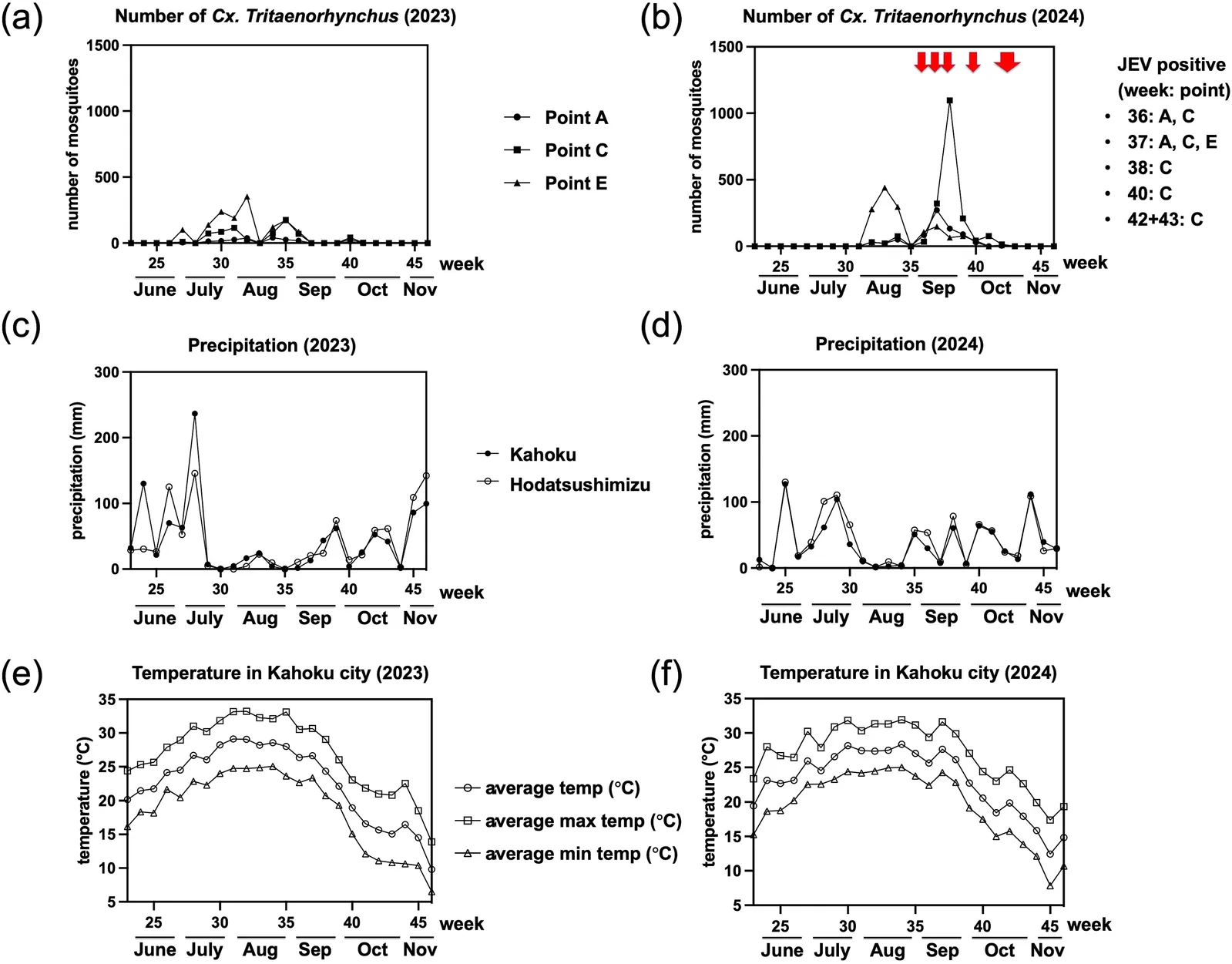
Collected number of *Cx. tritaeniorhynchus* at each sampling site and week, and precipitation in Kahoku city and Hodatsushimizu town, and temperature in Kahoku city. The number of *Cx. tritaeniorhynchus* in (a) 2023 and (b) 2024, the precipitation in (c) 2023 and (d) 2024, and the average temperature in (e) 2023 and (f) 2024. Red arrow representing JEV positivity in mosquito pool. (For interpretation of the references to colour in this figure legend, the reader is referred to the web version of this article.)

In total, 194, 676 and 1434 female *Cx. tritaeniorhynchus* were captured at points A, C, and E in 2023, whereas 716, 1925 and 1470 were captured at the same sites in 2024 (Table 1). The peak number of mosquitoes captured is September at points A and C in 2024, in August 2023, and point E in 2024 (Fig. 2a, b). Each year, the numbers of *Cx. tritaeniorhynchus* gradually increased in summer, peaking between August and September (Fig. 2a, b). The rainfall increased during the rainy season in July, whereas the highest temperatures were observed in August (Fig. 2c-f).

**Table 1:** Weekly captured number of *Cx*. *tritaeniorhynchus* captured in each point.

Site	Year	June				July				August					September				October						November		Total
		w23	w24	w25	w26	w27	w28	w29	w30	w31	w32	w33	w34	w35	w36	w37	w38	w39	w40	w41	w42	w43	w44		w45	w46	
A	2023	0	NT	0	NT	4	NT	13	16	26	38	NT	41	26	18	NT	NT	NT	10	1	1	0	0	0		0	194
	2024	0	0	0	NT	NT	NT	NT	NT	NT	30	22	50	NT	84*	272*	133	91	29	0	4	1	0	0		0	716
C	2023	0	NT	0	NT	9	NT	73	86	115	25	NT	77	176	70	NT	NT	NT	42	3	0	0	0	0		0	676
	2024	0	0	0	NT	NT	NT	NT	NT	NT	31	22	75	NT	34*	322*	1097*	209	43*	77	14**	1**	0	0		0	1925
E	2023	0	NT	0	NT	102	NT	138	238	190	353	NT	122	173	87	NT	NT	NT	28	1	1	1	0	0		0	1434
	2024	0	2^#^	1^#^	NT	NT	NT	NT	NT	NT	279	441	297	NT	108	149*	65	77	42	0	8	1	0	0		0	1470

W followed by a number indicating the epidemiological week (e.g., w23 = week 23). * indicating samples tested positive for JEV; ** tested in the same pool and positive for JEV. NT: sampling not conducted due to adverse weather conditions. ^#^ RT-PCR not tested due to sample loss.

### 3.2 Phylogenetic analysis from Flavivirus-positive pools

**Fig. 3:**
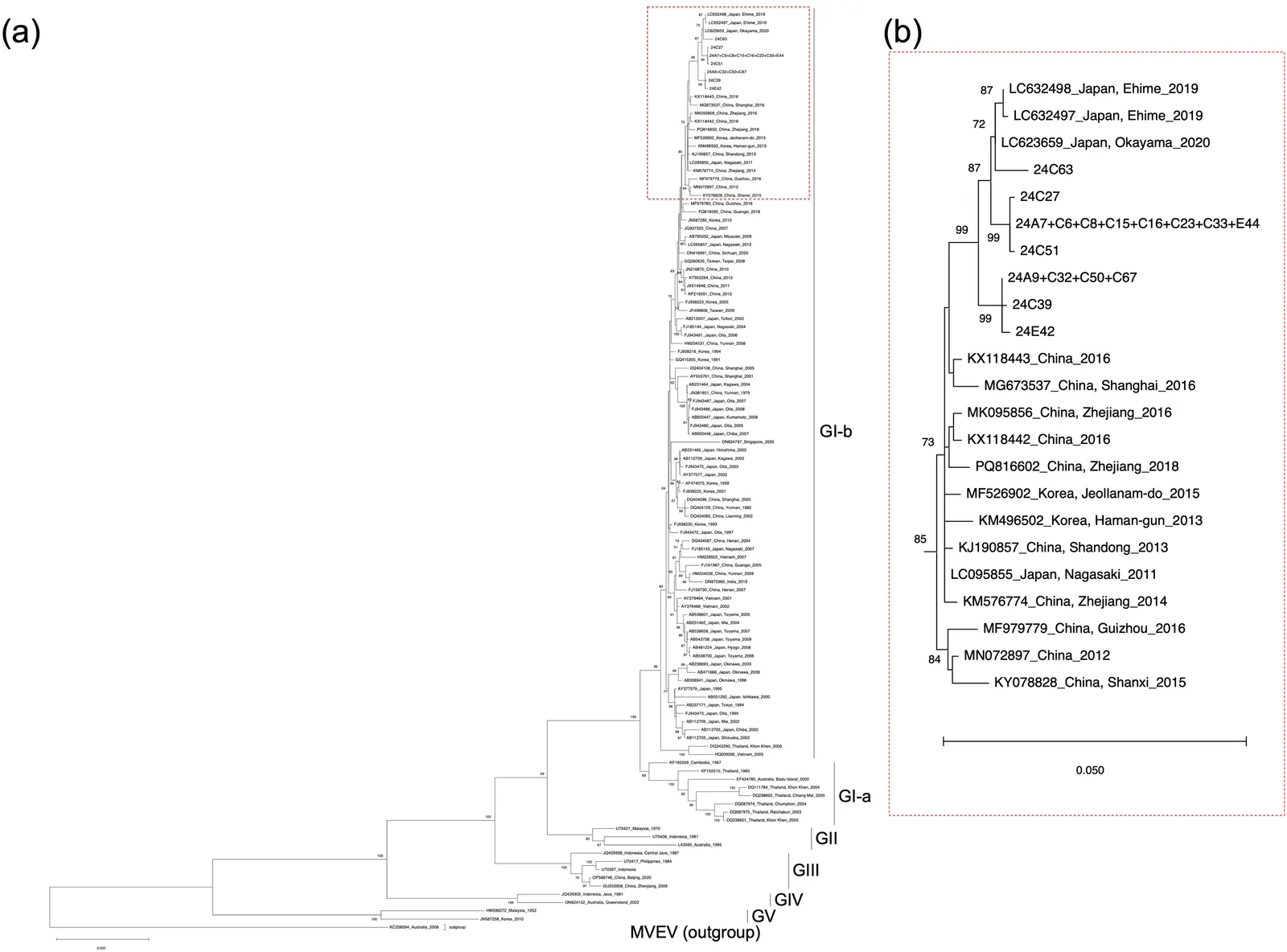
Phylogenetic tree of JEV from *Cx. tritaeniorhynchus*. (a) It constructed based on the E protein gene using the maximum-likelihood method. Murray Valley encephalitis virus (MVEV) included as an outgroup. (b) Enlarged view of the region indicated by the red dashed box in (a). All JEV-positive samples collected in 2024. The alphabets in the sample names indicating the collection site, and the specific collection dates for the sequences shown as follows: 24C63 (1 Oct), 24C27 (18 Sep), 24A7 (4 Sep), C6 (4 Sep), C8 (10 Sep), C15 (10 Sep), C16 (10 Sep), C23 (18 Sep), C33 (18 Sep), E44 (10 Sep), 24C51 (18 Sep), 24A9 (10 Sep), C32 (18 Sep), C50 (18 Sep), C67 (18 and 22 Oct), C39 (18 Sep), E42 (10 Sep). Bootstrap values ≥60% shown. (For interpretation of the references to colour in this figure legend, the reader is referred to the web version of this article.)

First, we performed RT-PCR targeting the Flavivirus NS5 region for screening. In 2023, three pools from point E were RT-PCR-positive, although no positive pools were detected at points A and C. In 2024, 2, 13, and 2 pools from points A, C, and E, respectively, were RT-PCR-positive (Table 2). To elucidate the genetic characteristics of flaviviruses detected by RT-PCR, we determined the nucleotide sequences from all NS5 positive samples (Fig. S1). In 2023, all sequences at point E were Culex flavivirus (CxFV), an insect-specific flavivirus, and all samples detected in 2024 were JEV (Table S1, Fig. S1-S2). Interestingly, several different strains were detected, even within the same day and at the trapping point (point C; 24C23, 24C32, and 24C39) (Table S1, Fig. 3, Fig. S3).

**Table 2:** RT-PCR positive sample pool number of *Cx. tritaeniorhynchus*.

Area (point)		2023			2024	
		RT-PCR positive (% positive pools)	MIR		RT-PCR positive (% positive pools)	MIR
A		0/10 (0)	0		2/26 (7.69)	2.79
C		0/24 (0)	0		13/67 (19.4)	6.75
E		3/50 (6.00)	2.09		2/52 (3.85)	1.36

Minimum infection rate (MIR) for flavivirus calculated as the number of infected pools per 1000 mosquitoes tested. The pool sizes ranging from 8 to 35 mosquitoes.

To examine the detailed genotype of JEV detected in 2024, we determined the sequences of the E protein. The sequences from points A, C, and E were all JEV GI-b-type in the phylogenetic analysis (Fig. 3, Fig. S3). Overall, all 17 sequences shared >98% identity. Phylogenetically, 24C63 clustered with those in Japan from 2019 to 2020 (Table S2), whereas the remaining 16 sequences formed distinct monophyletic clades that are clearly separated from the other reference sequences (Fig. 3, Fig. S3).

### 3.3 GLMM analysis of JEV positivity

The GLMM revealed that mosquito numbers had a significant positive effect on the infection with a coefficient estimate of 1.00 (SE = 0.33, *P* = 0.002) (Table 3). In contrast, no significant effect were observed for earthquake (Estimate = 4.97, SE = 3.01, *P* = 0.098), average temperature (Estimate =0.162, SE =0.353, *P* = 0.647), or precipitation (Estimate = −0.283, SE = 0.445, *P* = 0.525) (Table 3).

**Table 3:** Binomial GLMM of the probability of JEV in *Cx. tritaeniorhynchus*.

	Estimate	SE	*z*-value	*P*-value
(Intercept)	−6.56	1.99	−3.29	0.00101
Earthquake	4.97	3.01	1.65	0.0981
Average temperature	0.162	0.353	0.457	0.647
Precipitation	−0.283	0.445	−0.635	0.525
Mosquito numbers	1.00	0.330	3.04	0.00237

Data from the present study (2023–2024), and a re-analysis of the raw data from a previous study (2010–2014) (Murakami et al., 2017). Parameter effect estimates in the logit scale. Site and year included as random effects.

## 4 Discussion

The epicenter of the Noto earthquake was located at the tip of the peninsula (Fig. 1a). Although the area near the epicenter was not accessible, our previous research areas were also affected by the earthquake. The maximum seismic intensity at all points was 5-upper during the earthquake, causing considerable damage at each location (Fig. 1b, c, Fig. S4). Accordingly, we investigated the influence of the surrounding environment and observed several changes before and after the earthquake. One notable environmental change near point E was the expansion of water-ponded areas owing to topological changes following the earthquake (Fig. S4b).

Prior to the earthquake, we had continued investigation of the JEV positive rate in pooled mosquitoes in Ishikawa prefecture. From 2010 to 2014, 0 to 10.8% positive pools were detected depending on the year and the points (Murakami et al., 2017). In the present study, pooled samples from 2024 showed 3.85%–19.4% positivity for JEV, whereas those from 2023 were all negative for JEV. Notably, the number of mosquitoes per pool in this study was smaller than that in a previous study (Murakami et al., 2017), making the higher positivity observed in 2024 particularly notable. Interannual variations in climatic conditions during the study period may have influenced JEV positivity. Indeed, based on empirical trends observed during our study period, the emergence of mosquito seemed to lag behind rainfall and tracked alongside rising temperature. In a previous study, JE cases and rainfall were found to be positively correlated (Impoinvil et al., 2013). Temperature can also influence mosquito vectors and amplification hosts (Hsu et al., 2008). Thus, climatic factors can modify mosquito habitats and proliferation environments, resulting in temporal and spatial changes in mosquito density, which may in turn affect the JEV transmission dynamics (Tu et al., 2021). With these factors taken together, we employed a GLMM in this study to comprehensively explore the influence of both the earthquake and climate conditions. In the analysis, mosquito abundance was significantly associated with JEV positivity, suggesting the higher probability of JEV occurrence in areas with high vector density. Although the association between the earthquake and JEV positivity did not reach statistical significance, a positive trend was observed, which may indicate an ecological influence associated with the earthquake. Given that JEV was only detected after the earthquake, the lack of statistical significance may partly reflect the limited duration of post-earthquake surveillance.

Drastic environmental changes caused by natural disasters can influence vector ecology (Watson et al., 2007). In 2024, the Noto earthquake caused substantial environmental changes, and JEV-positive mosquitoes were subsequently detected. Mosquitoes and disaster-affected areas were investigated in a previous study. Several mosquito species including *Cx*. *tritaeniorhynchus* larvae and adults were captured in a post-disaster area after the Great East Japan Earthquake in 2011 (Watanabe et al., 2012). In addition, mosquito-borne infectious diseases, such as malaria (Saenz et al., 1995) and Zika virus disease (Reina Ortiz et al., 2017) have been reported to increase in disaster-affected areas, where altered hydrology and the formation of standing water bodies create favorable conditions for mosquito breeding and survival. Previous studies have shown that natural disasters can influence insect community structure. For example, increased standing water following hurricanes in Miami during 2016–2018 resulted in changes in mosquito species composition and abundance (Moise et al., 2024), and shifts in insect communities were observed after the 1999 Chi-Chi earthquake in Taiwan (Maa et al., 2006). Indeed, in the present study area, land subsidence and subsequent geophysical changes have led to the partial submergence of waterfront areas near trapping point E that were previously above ground (Fig. S4b). Cracks in roads and natural ground surfaces also accumulate rainwater after precipitation, thereby increasing the number of potential mosquito breeding sites (Fig. 1b, c, Fig. S4a, c). In addition, post-earthquake reconstruction and associated land-use changes have been shown to influence mosquito species composition (Samson et al., 2015), highlighting the importance of continued mosquito monitoring.

To further evaluate the spread and genetic characteristics of JEV detected in 2024, we conducted phylogenetic analysis of samples from points A, C, and E based on the envelope (E) protein sequence (Solomon et al., 2003; Sumiyoshi et al., 1987). The genotype of the JEV detected from all points was GI-b, which is consistent with its geographical distribution in the temperate climate of East Asia (Schuh et al., 2013). A phylogenetic analysis of the sequences revealed at least three distinct clades (Fig. 3 and Fig. S3), suggesting multiple independent introductions of the virus from different source populations and/or at different times in the region. Regarding seasonality, JE transmission typically peak in summer and autumn in temperate climates (Amicizia et al., 2018), which coincides with the detection of JEV in *Cx. tritaeniorhynchus* in September and October, respectively (Fig. 2b).

In comparison with reference JEV sequences, our results suggest that temporal separation may contribute more strongly than geographic distance to genetic divergence. Phylogenetically, the sequences detected in this study are more closely related to those from the 2010s to 2020s in various regions than to the sequences reported in Ishikawa Prefecture (2000) and in the neighboring Toyama Prefecture in the 2000s (Fig. 3, Fig. S3, Table S2). This is consistent with a previous Bayesian phylogeographic study of JEV demonstrating phylogenetic structure reflecting long-term evolutionary history with distinct divergence times among lineages, which supports the importance of temporal factors in shaping JEV genetic diversity (Schuh et al., 2013). One factor contributing to the temporal similarity may be the capacity of the vector for long-distance dispersal. *Cx. tritaeniorhynchus* exhibits substantial flight capacity, with meta-analytic data indicating an average maximum flight distance exceeding 2 km under self-powered conditions, highlighting its potential for local and regional dispersal as a vector of JEV (Verdonschot and Besse-Lototskaya, 2014). Moreover, direct evidence indicates that pathogen-transmitting mosquitoes can be transported over hundreds of kilometers by high-altitude wind currents, as demonstrated by aerial collections and atmospheric modeling in the Sahel region of sub-Saharan Africa (Huestis et al., 2019). To support this, phylogenetic analyses have suggested that some *Cx. tritaeniorhynchus* specimens trapped in western Japan during summer belong to continental lineages (Arai et al., 2022). Meteorological analyses further indicated that atmospheric flows prior to capture were consistent with wind-borne transport from mainland Asia across the East China Sea. Thus, *Cx. tritaeniorhynchus* carrying JEV may have been introduced into the study area from neighboring regions via wind-assisted dispersal.

In the present study, only GI-b was detected; however, sporadic detection of other genotypes, including GV, which continues to be detected in East Asia and remains a focus of ongoing genetic surveillance efforts (Takhampunya et al., 2011; Li et al., 2011; Yun et al., 2025). This highlights the need for continued genetic surveillance to monitor potential changes in JEV genotype distribution. Years with low vaccine coverage may have contributed to the increased circulation of the virus. In Japan, vaccination against Japanese encephalitis was introduced in 1954 (Takeshita et al., 2014). However, concerns regarding the adverse events associated with the previously used mouse brain-derived vaccine led to the suspension of active recommendations in 2005, resulting in a marked decline in vaccination coverage between 2005 and 2009 (Nanishi et al., 2019). Given these considerations, attention should be paid to the potential future increase in JE cases.

Viral interference between co-circulating viruses is another factor that may influence viral transmission in host-virus systems (Vasilakis and Tesh, 2015). However, data on the effects of CxFV on mosquitoes are limited, and a previous cell-based study reported that persistent CxFV infection did not prevent JEV infection, but reduced its replication efficacy (Kuwata et al., 2015). Although CxFV was detected in 2023 samples, its influence on JEV circulation may have been limited. Further studies are needed to fully elucidate the role of viral interference and its effect on JEV positivity in mosquito populations.

As a limitation, mosquito collection could not be conducted for several weeks in both years because of adverse weather conditions. Nevertheless, actual detection of JEV in the present study, combined with the high seroprevalence in recent swine populations (National Institute of Infectious Diseases, 2024) and wild boars (Komiya et al., 2019; Yazawa et al., 2025), unvaccinated populations including travelers remain at risk, particularly during the summer to autumn seasons.

## 5 Conclusion

We demonstrated the presence of JEV-positive mosquitoes in disaster-affected areas and its surrounding region following the Noto earthquake in 2024. Overall, the findings in the present study underscore the potential for continued environmental circulation of JEV following large-scale earthquakes that may alter vector-host dynamics, and highlight the importance of sustained entomological surveillance in disaster-affected regions.

## CRediT authorship contribution statement

**Kota Mochizuki:** Writing – review & editing, Writing – original draft, Visualization, Methodology, Investigation, Formal analysis, Data curation. **Yosaburo Oikawa:** Writing – review & editing, Conceptualization. **Manabu Murakami:** Writing – review & editing, Writing – original draft, Validation, Supervision, Resources, Project administration, Methodology, Investigation, Funding acquisition, Formal analysis, Conceptualization.
