# Supplementary material for: Detection of Japanese encephalitis virus in Culex tritaeniorhynchus following the 2024 Noto Peninsula earthquake, Japan
Source: Parasite Epidemiol Control. 2026 Aug 23;34:e00539. doi: 10.1016/j.parepi.2026.e00539 (PMC13546856; doi:10.1016/j.parepi.2026.e00539)
Supplement: Supplementary file 1 [file mmc1.docx]

Table S1 List of samples and GenBank accession numbers for NS5 and E protein sequences.

| Sample | Date | NS5 | |  | E protein | |
| --- | --- | --- | --- | --- | --- | --- |
|  |  | Species, genotype | Accession |  | Species, genotype | Accession |
| 23E10 | 23 July 2023 | CxFV | LC915187 |  | - | - |
| 23E41 | 30 Aug 2023 | CxFV | LC915188 |  | - | - |
| 23E42 | 30 Aug 2023 | CxFV | - |  | - | - |
| 24A7 | 4 Sep 2024 | JEV, GI | LC915189 |  | JEV, GI-b | LC915194 |
| 24A9 | 10 Sep 2024 | JEV, GI | - |  | JEV, GI-b | LC934768 |
| 24C6 | 4 Sep 2024 | JEV, GI | - |  | JEV, GI-b | LC934769 |
| 24C8 | 10 Sep 2024 | JEV, GI | - |  | JEV, GI-b | LC934770 |
| 24C15 | 10 Sep 2024 | JEV, GI | - |  | JEV, GI-b | LC934771 |
| 24C16 | 10 Sep 2024 | JEV, GI | - |  | JEV, GI-b | LC934772 |
| 24C23 | 18 Sep 2024 | JEV, GI | - |  | JEV, GI-b | LC934773 |
| 24C27 | 18 Sep 2024 | JEV, GI | - |  | JEV, GI-b | LC934774 |
| 24C32 | 18 Sep 2024 | JEV, GI | - |  | JEV, GI-b | LC934775 |
| 24C33 | 18 Sep 2024 | JEV, GI | - |  | JEV, GI-b | LC934776 |
| 24C39 | 18 Sep 2024 | JEV, GI | LC915190 |  | JEV, GI-b | LC915195 |
| 24C50 | 18 Sep 2024 | JEV, GI | - |  | JEV, GI-b | LC934777 |
| 24C51 | 18 Sep 2024 | JEV, GI | - |  | JEV, GI-b | LC934778 |
| 24C63 | 1 Oct 2024 | JEV, GI | LC915191 |  | JEV, GI-b | LC915196 |
| 24C67 | 18, 22 Oct 2024 | JEV, GI | LC915192 |  | JEV, GI-b | LC915197 |
| 24E42 | 10 Sep 2024 | JEV, GI | LC915193 |  | JEV, GI-b | LC915198 |
| 24E44 | 10 Sep 2024 | JEV, GI | - |  | JEV, GI-b | LC934779 |

-; identical NS5 sequences (see Fig. S1) deposited for a single representative.

Fig. S1 Alignment of all NS5 sequences detected from ***Cx. tritaeniorhynchus*. Same color representing sequences with 100% identity. Two *Culex flavivirus* sequences (blue arrowheads) and five JEV sequences (red arrowheads), were determined and deposited in the Genbank (accession numbers in Table S1).**

Fig. S2 Phylogenetic tree of *Culex flavivirus* from ***Cx. tritaeniorhynchus,*** constructed based on the NS5 gene using the neighbor-joining method. JEV sequences including samples detected in the present study included as the outgroup. Red arrow indicating sample sequences detected in 2023. Bootstrap values $\geq$60% shown.

Fig. S3 Phylogenetic tree of JEV from ***Cx. tritaeniorhynchus***. It constructed based on the E protein gene using the neighbor-joining method. Murray Valley encephalitis virus (MVEV) included as an outgroup. (b) Enlarged view of the region indicated by the red dashed box in (a). All positive samples collected in 2024. Bootstrap values $\geq$60% shown.

Fig. S4 Topographic changes caused by the earthquake. (a) Vicinity of point A, showing large cracks on the road (red arrowhead). (b) Water-front area near point E before (2023) and after (2024) the earthquake. The inundated area increasing as a result of ground deformation (blue arrow). (c) Vicinity of point C, showing collapsed riverbank and partially damaged guardrail.
