# Supplementary material for: Detection of Japanese encephalitis virus in Culex tritaeniorhynchus following the 2024 Noto Peninsula earthquake, Japan
Source: Parasite Epidemiol Control. 2026 Aug 23;34:e00539. doi: 10.1016/j.parepi.2026.e00539 (PMC13546856; doi:10.1016/j.parepi.2026.e00539)
Supplement: Supplementary file 2 [file mmc2.docx]

Table S2 Details of the JEV sequences used in this study.

| Isolate | Accession No. | Country/Location | Year | Species/genotype | Host | Sources/References |
| --- | --- | --- | --- | --- | --- | --- |
| IM-OI34 | LC632498 | Japan/Ehime | 2019 | GI-b | Tick | PMID: 35405649 |
| IM-OI27 | LC632497 | Japan/Ehime | 2019 | GI-b | Tick | PMID: 35405649 |
| JEV/Hu/Okayama/NIID13/2020 | LC623659 | Japan/Okayama | 2020 | GI-b | Human | PMID: 35405649 |
| LYG-3 | KX118443 | China/- | 2016 | GI-b | Mosquito | Direct submission |
| HP4A_16-7-H-Cut-C-5-2 | MG673537 | China/Shanghai | 2016 | GI-b | Mosquito | PMID: 30626442 |
| ZY-YW-4-16 | MK095856 | China/Zhejiang | 2016 | GI-b | Mosquito | Direct submission |
| LYG-2 | KX118442 | China/- | 2016 | GI-b | Mosquito | Direct submission |
| ZJ-01 | PQ816602 | China/Zhejiang | 2018 | GI-b | Swine | Direct submission |
| K15JN03 | MF526902 | Korea/Jeollanam-do | 2015 | GI-b | Mosquito | Direct submission |
| K13GN46 | KM496502 | Korea/Haman-gun | 2013 | GI-b | Mosquito | Direct submission |
| RC57/S/CHN/13 | KJ190857 | China/Shandong | 2013 | GI-b | Mosquito | PMID: 24809635 |
| JaNP97-11 | LC095855 | Japan/Nagasaki | 2011 | GI-b | Swine | PMID: 27433127 |
| ZJ14-06 | KM576774 | China/Zhejiang | 2014 | GI-b | Mosquito | Direct submission |
| GZDJ1609 | MF979779 | China/Guizhou | 2016 | GI-b | Mosquito | PMID: 29058107 |
| SaX12-35 | MN072897 | China/- | 2012 | GI-b | Mosquito | Direct submission |
| SXYC1548 | KY078828 | China/Shanxi | 2015 | GI-b | Mosquito | PMID: 28592296 |
| GZDJ1648 | MF979780 | China/Guizhou | 2016 | GI-b | Mosquito | PMID: 29058107 |
| GX-02 | PQ816595 | China/Guangxi | 2018 | GI-b | Mosquito | Direct submission |
| A10.881 | JN587260 | Korea/- | 2010 | GI-b | Mosquito | PMID: 21943222 |
| LN0702 | JQ937353 | China/- | 2007 | GI-b | Mosquito | PMID: 2406905 |
| JEV/Bo/Miyazaki/1/2009 | AB795032 | Japan/Miyazaki | 2009 | GI-b | Bovine | PMID: 23885004 |
| JaNP95-12 | LC095857 | Japan/Nagasaki | 2012 | GI-b | Swine | PMID: 27433127 |
| SCLS-202005 | ON416991 | China/Sichuan | 2020 | GI-b | Swine | PMID: 35672440 |
| TPC0806c | GQ260635 | Taiwan/Taipei | 2008 | GI-b | Mosquito | PMID: 20409392 |
| ZJ10-45 | JN216870 | China/- | 2010 | GI-b | Mosquito | Direct submission |
| SCYA201201 | KT952294 | China/- | 2012 | GI-b | Swine | Direct submission |
| 3XG001 | JX514948 | China/- | 2011 | GI-b | Mosquito | Direct submission |
| 12-YJ038 | KP216591 | China/- | 2012 | GI-b | Mosquito | Direct submission |
| K05-GS | FJ938223 | Korea/- | 2005 | GI-b | Mosquito | PMID: 20546562 |
| YL2009-2 | JF499806 | Taiwan/- | 2009 | GI-b | Mosquito | PMID: 22172307 |
| JEV/eq/Tottori/2003 | AB213007 | Japan/Tottori | 2003 | GI-b | Equine | PMID: 16598178 |
| JaNAr07-04 | FJ185144 | Japan/Nagasaki | 2004 | GI-b | Mosquito | PMID: 19264633 |
| 06P152 | FJ943481 | Japan/Oita | 2006 | GI-b | Swine | PMID: 20035900 |
| FQ24M-08 | HM204531 | China/Yunnan | 2008 | GI-b | Mosquito | Direct submission |
| K94A07 | FJ938216 | Korea/- | 1994 | GI-b | Mosquito | PMID: 20546562 |
| JE-91 | GQ415355 | Korea/- | 1991 | GI-b | Mosquito | PMID: 19776238 |
| SH05-24 | DQ404108 | China/Shanghai | 2005 | GI-b | Mosquito | PMID: 17325361 |
| SH-101 | AY555761 | China/Shanghai | 2001 | GI-b | Mosquito | Direct submission |
| JEV/sw/Kagawa/35/2004 | AB231464 | Japan/Kagawa | 2004 | GI-b | Swine | PMID: 17872529 |
| YN79Bao83 | JN381851 | China/Yunnan | 1979 | GI-b | Mosquito | PMID: 21697481 |
| 07P127 | FJ943487 | Japan/Oita | 2007 | GI-b | Swine | PMID: 20035900 |
| 08P37 | FJ943488 | Japan/Oita | 2008 | GI-b | Swine | PMID: 20035900 |
| JE/sw/Kumamoto/104/2006 | AB920447 | Japan/Kumamoto | 2006 | GI-b | Swine | Direct submission |
| 05P75 | FJ943480 | Japan/Oita | 2005 | GI-b | Swine | PMID: 20035900 |
| JE/sw/Chiba/150/2007 | AB920448 | Japan/Chiba | 2007 | GI-b | Swine | Direct submission |
| SG/EHI-MS_CT261_Nov_2020 | ON804797 | Singapore/Singapore | 2020 | GI-b | Mosquito | PMID: 36560666 |
| JEV/sw/Hiroshima/25/2002 | AB231465 | Japan/Hiroshima | 2002 | GI-b | Swine | PMID: 17872529 |
| JEV/sw/Kagawa/24/2002 | AB112706 | Japan/Kagawa | 2002 | GI-b | Swine | PMID: 17872529 |
| 03P113 | FJ943475 | Japan/Oita | 2003 | GI-b | Swine | PMID: 20035900 |
| JaNAr0102 | AY377577 | Japan/- | 2002 | GI-b | Mosquito | Direct submission |
| KV1899 | AF474075 | Korea/- | 1999 | GI-b | Swine | Direct submission |
| K01-GN | FJ938220 | Korea/- | 2001 | GI-b | Mosquito | PMID: 20546562 |
| SH03-103 | DQ404096 | China/Shanghai | 2003 | GI-b | Mosquito | PMID: 17325361 |
| YN82-BN8219 | DQ404129 | China/Yunnan | 1982 | GI-b | Mosquito | PMID: 17325361 |
| LN02-102 | DQ404085 | China/Liaoning | 2002 | GI-b | Mosquito | PMID: 17325361 |
| K93A07 | FJ938230 | Korea/- | 1993 | GI-b | Mosquito | PMID: 20546562 |
| 97P82 | FJ943472 | Japan/Oita | 1997 | GI-b | Swine | PMID: 20035900 |
| HN04-11 | DQ404087 | China/Henan | 2004 | GI-b | Mosquito | PMID: 17325361 |
| JaNAr06-07 | FJ185143 | Japan/Nagasaki | 2007 | GI-b | Mosquito | PMID: 19264633 |
| 07VN310 | HM228922 | Vietnam/- | 2007 | GI-b | Mosquito | Direct submission |
| GX0519 | FJ161967 | China/Guangxi | 2005 | GI-b | Mosquito | Direct submission |
| LX10P-09 | HM204528 | China/Yunnan | 2009 | GI-b | Human | Direct submission |
| JEV/GIII | ON875960 | India/- | 2019 | GI-b | Mosquito | Direct submission |
| HEN0701 | FJ156730 | China, Henan | 2007 | GI-b | Swine | Direct submission |
| VN88 | AY376464 | Vietnam/- | 2001 | GI-b | Swine | Direct submission |
| VN105 | AY376468 | Vietnam/- | 2002 | GI-b | Mosquito | Direct submission |
| Mo/Toyama/1018c/2005 | AB538601 | Japan/Toyama | 2005 | GI-b | Mosquito | PMID: 21540378 |
| JEV/sw/Mie/34/2004 | AB231462 | Japan/Mie | 2004 | GI-b | Swine | PMID: 17872529 |
| Mo/Toyama/2347c/2007 | AB538658 | Japan/Toyama | 2007 | GI-b | Mosquito | PMID: 21540378 |
| Mo/Toyama/3133c/2009 | AB543738 | Japan/Toyama | 2009 | GI-b | Mosquito | PMID: 21540378 |
| - | AB481224 | Japan/Hyogo | 2008 | GI-b | Swine | Direct submission |
| Mo/Toyama/2757c/2008 | AB538700 | Japan/Toyama | 2008 | GI-b | Mosquito | PMID: 21540378 |
| JEV/sw/Okinawa/285/2003 | AB238693 | Japan/Okinawa | 2003 | GI-b | Swine | PMID: 17872529 |
| JEV/sw/Okinawa/154/2008 | AB471666 | Japan/Okinawa | 2008 | GI-b | Swine | Direct submission |
| JEV/wb/Okinawa/1998 | AB306941 | Japan/Okinawa | 1998 | GI-b | Swine | PMID: 18362415 |
| 95-167 | AY377579 | Japan/- | 1995 | GI-b | Swine | Direct submission |
| Ishikawa | AB051292 | Japan/Ishikawa | 2000 | GI-b | Mosquito | PMID: 11056567 |
| JaTAn 1/94 | AB237171 | Japan/Tokyo | 1994 | GI-b | Swine | PMID: 17872529 |
| 99P103 | FJ943473 | Japan/Oita | 1999 | GI-b | Swine | PMID: 20035900 |
| JEV/sw/Mie/41/2002 | AB112709 | Japan/Mie | 2002 | GI-b | Swine | PMID: 17872529 |
| JEV/sw/Chiba/88/2002 | AB112705 | Japan/Chiba | 2002 | GI-b | Swine | PMID: 17872529 |
| JEV/sw/Shizuoka/33/2002 | AB112703 | Japan/Shizuoka | 2002 | GI-b | Swine | PMID: 17872529 |
| JE_KK_1116 | DQ343290 | Thailand/Khon Khen | 2005 | GI-b | Unknown | Direct submission |
| CT-MO-P7 | HQ009266 | Vietnam/- | 2005 | GI-b | Swine | Direct submission |
| M859/Cambodia/1967/Mosquito | KF192509 | Cambodia/- | 1967 | GI-a | Mosquito | PMID: 24009790 |
| KE-93-83 | KF192510 | Thailand/- | 1983 | GI-a | Mosquito | PMID: 24009790 |
| TS00 | EF434785 | Australia/Badu Island | 2000 | GI-a | Swine | Direct submission |
| JE_KK_80 | DQ111784 | Thailand/Khon Khen | 2004 | GI-a | Unknown | Direct submission |
| JE_CM_1196 | DQ238602 | Thailand/Chiang Mai | 2005 | GI-a | Unknown | Direct submission |
| JE_CP_49 | DQ087974 | Thailand/Chumphon | 2004 | GI-a | Unknown | Direct submission |
| JE_RT_36 | DQ087975 | Thailand/Ratchaburi | 2003 | GI-a | Mosquito | Direct submission |
| JE_KK_577 | DQ238601 | Thailand/Khon Khen | 2005 | GI-a | Unknown | Direct submission |
| WTP-70-22 | U70421 | Malaysia/- | 1970 | G-II | Mosquito | Direct submission |
| JKT5441/Indonesia/1981/Mosquito | U70406 | Indonesia/- | 1981 | GII | Mosquito | Direct submission |
| FU | L43565 | Australia/- | 1995 | GII | Human | PMID: 8816682 |
| JKT27-087 | JQ429308 | Indonesia/Central Java | 1987 | GIII | Mosquito | PMID: 23590316 |
| PhAn1242/the Philippines/1984/Pig/serum | U70417 | Philippines | 1984 | GIII | Swine | Direct submission |
| Indonesia/Indonesia/Mosquito | U70397 | Indonesia/- | - | GIII | Unknown | Direct submission |
| Beijing/2020-1 | OP588746 | China/Beijing | 2020 | GIII | Mosquito | Direct submission |
| Mo/Zhenjiang/1/2009 | GU253958 | China/Zhenjiang | 2009 | GIII | Mosquito | Direct submission |
| JKT7089 | JQ429309 | Indonesia/Java | 1981 | GIV | Mosquito | PMID: 23590316 |
| JEV/sw-22-00722-11/Qld/2022 | ON624132 | Australia/Queensland | 2022 | GIV | Swine | Direct submission |
| Muar | HM596272 | Malaysia/- | 1952 | GV | Human | PMID: 21352956 |
| 10-1827 | JN587258 | Korea/- | 2010 | GV | Mosquito | PMID: 21943222 |
| K62017 | KC206094 | Australia/Western | 2006 | MVEV | mosquito | PMID: 26600318 |

Murray Valley encephalitis virus: MVEV. Unpublished data shown as Direct submission.
